# Development of Virus-like Particle Plant-Based Vaccines against Avian H5 and H9 Influenza A Viruses

**DOI:** 10.3390/vetsci11020093

**Published:** 2024-02-18

**Authors:** Ola A. Elbohy, Munir Iqbal, Janet M. Daly, Stephen P. Dunham

**Affiliations:** 1 School of Veterinary Medicine and Science, University of Nottingham, Sutton Bonington Campus, Loughborough LE12 5RD, UK; ola.elbohy2@nottingham.ac.uk; 2 Department of Virology, Faculty of Veterinary Medicine, Mansoura University, Mansoura 35516, Egypt; 3 Avian Influenza Group and Newcastle Disease, The Pirbright Institute, Woking GU24 0NF, UK; munir.iqbal@pirbright.ac.uk

**Keywords:** virus-like particles, *Nicotiana benthamiana*, ELISpot, pseudotyped virus neutralisation test, influenza, plant expression

## Abstract

**Simple Summary:**

Avian influenza virus (AIV) is a highly infectious viral disease, some subtypes of which (e.g., H5 and H9) cause high mortality in poultry and can be transmitted to humans. AIV continues to cause severe losses despite the availability of commercial vaccines. Haemagglutinin (HA) is the main antigen of the virus and neutralising antibodies are elicited against it upon infection. Virus-like particles (VLPs) mimic the shape of the virus but are devoid of the inner genetic material, so they are non-infectious but provoke a strong immune response. The aim of this project was to produce cheap, safe and effective plant-based vaccines by producing H9 and H5 VLPs in *Nicotiana benthamiana*. A transmission electron microscope (TEM) was used to visualise VLPs derived from plant extracts. Mice were initially immunised with multiple doses of the H5 and H9 VLPs to confirm that they elicited robust antibody and T-cell immune responses. It was then demonstrated that a single dose of H5 VLPs given to chickens stimulated an antibody response that could neutralise virus infectivity. These results show that plant-based vaccines are a promising alternative to traditional vaccines, especially in developing countries, and further development is desirable.

**Abstract:**

Avian influenza A virus (AIV) is a significant cause of mortality in poultry, causing substantial economic loss, particularly in developing countries, and has zoonotic potential. For example, highly pathogenic avian influenza (HPAI) viruses of the H5 subtype have been circulating in Egypt for around two decades. In the last decade, H5N1 viruses of clade 2.2.1 have been succeeded by the antigenically distinct H5N8 clade 2.3.4.4b viruses. Furthermore, H9N2 viruses co-circulate with the H5N8 viruses in Egyptian poultry. It is widely recognised that effective vaccination against IAV requires a close antigenic match between the vaccine and viruses circulating in the field. Therefore, approaches to develop cost-effective vaccines that can be rapidly adapted to local virus strains are required for developing countries such as Egypt. In this project, the haemagglutinin (HA) proteins of Egyptian H5 and H9 viruses were expressed by transient transfection of plants (*Nicotiana benthamiana*). The formation of virus-like particles (VLPs) was confirmed by transmission electron microscopy. Mice were immunised with four doses of either H5 or H9 VLPs with adjuvant. Antibody and cellular immune responses were measured against the corresponding recombinant protein using ELISA and enzyme-linked immunosorbent assay (ELISpot), respectively. Chickens were immunised with one dose of H5 VLPs, eliciting HA-specific antibodies measured by ELISA and a pseudotyped virus neutralisation test using a heterologous H5 HA. In conclusion, plant-based VLP vaccines have potential for producing an effective vaccine candidate within a short time at a relatively low cost.

## 1. Introduction

Avian influenza is a contagious viral disease affecting poultry, some strains of which have zoonotic potential. Avian influenza viruses (AIVs) belong to the family *Orthomyxoviridae* and genus influenza A virus (IAV) [1]. Waterfowl are the major reservoir for AIVs; virus mutation and recombination are a major risk for the emergence of new virus strains, which may lead to zoonotic transmission to humans, with the potential for pandemic spread [2]. AIVs are enveloped and within this envelope are embedded haemagglutinin (HA) and neuraminidase (NA) glycoprotein spikes. The viral core contains the virus genome composed of eight single-stranded negative-sense RNA genome segments [3]. According to the pathogenicity, AIV is classified into highly pathogenic AIV (HPAIV) and low-pathogenicity AIV (LPAIV) [4]. To date, there are 16 subtypes of HA and 9 subtypes of NA that have been recorded in poultry and wild birds [5]. Some subtypes of H5Nx are classified as HPAIV, while H9Nx are classified as LPAIV. Chickens with LPAIV infections suffer from mild to severe respiratory signs, decreased egg production, increased morbidity and up to 20% mortality [6]. Lesions may also involve subcutaneous haemorrhages, cyanosis of the head and comb and oedema in severe cases. HPAI viruses can cause more serious illness in poultry, with up to 100% mortality [7]. H5N8 and H9N2 were the dominant AIV subtypes infecting poultry in many countries, including Egypt, between 2014 and 2020 [8,9]. Clade 2.3.4.4b of H5N8 AIVs have been reported in wild birds in Egypt since 2016 and have since spread to birds in live poultry markets [10]. Viruses belonging to the H9N2 G1-like lineage were first detected in Egyptian farms in 2010 and became endemic in different regions in Egypt [11]. Co-circulation of H5N8 and H9N2 in live poultry markets increases the risk of novel reassortment and emergence of viruses with zoonotic potential [9]. Continuous surveillance and monitoring of AIVs in Egypt is therefore highly recommended to control AIV outbreaks.

Control strategies for AIV vary depending on geographical location and disease status. While some countries, including the UK and USA, rely on strict biosecurity and culling of infected birds in the event of an outbreak, many countries use vaccination to protect poultry from severe disease and decrease virus shedding post-infection, in addition to biosecurity [12]. However, AIV has a high mutation rate, which can lead to an increasing mismatch between vaccinal and field strains, resulting in a loss of disease control as vaccine effectiveness declines [13]. The World Organisation for Animal Health (WOAH) has tasked experts to assess the current situation and review recommendations for controlling virus spread. The latest recommendations call for enhanced biosecurity levels and maintenance of good hygiene practices on farms. They also have called for enhanced international collaborations and sharing of sequences [14]. In response to WOAH recommendations, the European Union applied rules around international movement of vaccinated animals and their products in March 2023 [15]. Indeed, WOAH recommends updating the vaccines every 2–3 years to maintain vaccine effectiveness [16]. Most current licensed AI vaccines in poultry are inactivated virus vaccines (to ensure safety), developed with adjuvants to enhance immune responses [7]. Although there are commercial vaccines available for AIV in poultry, these viruses continue to cause significant economic losses throughout the world. Poultry are a major source of animal protein, and the worldwide chicken population comprises over 20 billion birds. Poultry provide a valuable supply of dietary protein and an essential source of income in less-resourced countries. In 2022, 67 countries reported to WOAH regarding outbreaks of HPAIV H5 that resulted in 131 million poultry losses due to culling or death. Moreover, 14 countries reported massive mortality patterns in wild birds due to an H5N1 clade 2.3.4.4b viral outbreak [14]. Availability of more effective and cheaper vaccines would enable more widespread protection of vulnerable poultry.

Plant molecular farming has recently emerged as a prominent field of applied biotechnology [17]. Recombinant proteins can be expressed using transient or stable plant-based expression systems. A transient expression system is preferred because of the speed of production and reduced costs [18]. Plant-based vaccines are stable, affordable to produce, can be rapidly adapted to local virus strains, and are suitable for production in countries with fewer resources [19,20,21]. However, recombinant subunit proteins elicit a poor immune response against influenza [22,23]. In contrast, virus-like particles (VLPs) mimic the structure of the virus but are devoid of the inner nucleic acid; therefore, VLPs are safe (non-infectious) and able to stimulate both cellular and humoral immunity [24,25]. VLPs consist of assembled repeats of structural proteins for optimal presentation to the antigen-presenting cells [26]. Previous studies reported that the VLP vaccine platform enhanced immune responses compared to the recombinant proteins due to proper antigen presentation to the immune cells [27,28,29].

The efficacy of a plant-based influenza quadrivalent VLP vaccine (QVLP) was tested in humans at 30 μg/dose and was found to be more protective than the quadrivalent inactivated influenza vaccine at 15 μg/dose [30]. The QVLP was tested and reached phase 3 of clinical trials, which confirmed the immunogenicity and safety of the vaccine [31]. A SARS-CoV-2 vaccine also showed promising results in a phase 3 clinical trial. Unfortunately, neither vaccine has reached the commercialisation stage thus far, as Medicago, the company developing the vaccine, ceased operations in February 2023. Nonetheless, Medicago reported that plant-based vaccines can be produced within 5 weeks, which is advantageous during the early stages of a pandemic, especially when compared to egg- and cell-based production platforms that require around 4–6 months [32]. They also reported that 1 kg of infiltrated plants could yield 30,000 doses of VLPs for chicken immunisation, which is affordable for low- and middle-income countries (LMICs).

In this project, plant-based VLP vaccines containing HA antigens from clade 2.3.4.4b H5N8 virus and H9N2 virus were produced in *Nicotiana benthamiana* using *Agrobacterium tumefaciens* and the immunogenicity of the VLPs tested was evaluated in mice and chickens.

## 2. Materials and Methods

### 2.1. Construction of Plant Expression Vectors

The DNA sequences encoding HA of clade 2.3.4.4b H5N8 strain A/chicken/Egypt/FL6/2018 (accession number: MH986133; hereafter referred to as H5) and G1 lineage H9N2 strain A/Egypt/ZU63/2016 (accession number: KY910830; hereafter referred to as H9) were obtained from GenBank. The nucleotide sequences encoding mature peptides of HA1 and HA2 were codon-optimised for expression in *N. benthamiana* and synthesised (GeneArt Gene Synthesis, ThermoFisher Scientific, Loughborough, UK) with an *AgeI* restriction site, Kozak sequence (AACA) and the signal peptide sequence of tobacco pathogenesis-related protein-1a (PR1a; GenBank accession no. X06930) for targeting the protein to the endoplasmic reticulum (ER) at the N-terminus and a stop codon and *XhoI* restriction site at the C-terminus. Expression cassettes provided in the pMAT vector were digested using *AgeI* and *XhoI* restriction enzymes and cloned into the pEAQ-HT shuttle vector, which was gifted by Prof. George Lomonossoff (John Innes Centre, Norwich, England, UK) [33,34]. The pEAQ-HT shuttle vectors were transformed by electroporation into the *Agrobacterium* strain GV3101 gifted by Dr Kostya Kanyuka (Rothamsted Research, Harpenden, UK).

### 2.2. Transient Expression of Virus-like Particles in Nicotiana Benthamiana Plants

The H5 and H9 VLPs were transiently expressed in *N. benthamiana* leaves using the syringe infiltration method [35]. *Agrobacterium* GV3101 containing H5 or H9 pEAQ-HT expression constructs were separately cultured in Luria–Bertani Lennox (LB) medium containing antibiotics (50 µg/mL kanamycin and 15 µg/mL rifampicin) for 20 h at 28 °C with shaking at 225 rpm. The bacteria were harvested (centrifugation at 4000× *g*, 5 min, room temperature) and resuspended in infiltration buffer (10 mM 2-N-morpholino ethane sulfonic acid (MES), 10 mM MgSO_4_, pH 5.6) supplemented with 0.1 mM acetosyringone. *Agrobacterial* cultures were diluted in infiltration buffer to a final optical density at 600 nm of 0.5. *Nicotiana benthamiana* plants (6 weeks old) were infiltrated using a syringe without a needle. The infiltrated leaves were harvested 7 days post-infiltration and stored at −80 °C. Plants were also infiltrated with *Agrobacteria* containing pEAQ-HT with no insert to provide lysates for use as a negative control for immune assays.

### 2.3. Extraction of Virus-like Particles

The infiltrated leaves (15 g) were ground under liquid nitrogen followed by homogenisation in VLP extraction buffer (50 mM Tris, 75 mM NaCl and 0.1% Triton X-100) (1:3 ratio; mass: volume) with a commercial blender and filtered using Mira cloth. The plant extracts were centrifuged at 10,000× *g*, 15 min, at 4 °C and filtered using 0.45 μm syringe filters. Protein concentrations of the plant extracts were determined by Bradford assay.

### 2.4. SDS-PAGE and Western Blot

The VLPs were mixed with 4x reducing Laemmli buffer and heated at 100 °C for 5 min. Treated VLPs were separated using 12% Mini-PROTEAN^®^ TGX™ Precast Protein Gels (Bio-Rad, Watford, UK) at reducing conditions. After electrophoresis, the gels were stained with SimplyBlue™ SafeStain (Thermo Fisher Scientific, UK). VLPs were transferred into Trans-Blot Turbo Mini 0.2 µm Nitrocellulose Transfer Packs (Bio-Rad, UK). The blotted membranes were incubated with a 5% (*w*/*v*) skimmed milk powder dissolved in a phosphate-buffered saline (PBS) buffer for an hour. Then, membranes were incubated with specific H9 (1:1000, Stratech, Ely, UK) or H5 antibodies (1:1000, Stratech, UK) at 4 °C overnight. Finally, the membranes were incubated with anti-rabbit IgG horseradish peroxidase (HRP) linked antibody (1:5000, Cell Signaling Technology, Leiden, The Netherlands). Enhanced chemiluminescent (ECL; Promega, Southampton, UK) detection reagents were applied to the membranes and the chemiluminescent signals were detected using ChemiDoc MP Imaging System (Bio-Rad, UK).

### 2.5. Purification of the VLPs 

The VLPs were purified using ultracentrifugation. The filtered supernatant containing either H5 or H9 VLPs was added on top of a freshly prepared density cushion consisting of 2 mL each of 30 and 70% sucrose. Ultracentrifugation was performed (32,000× *g*, 3 h, 4 °C; Hitachi Ultra-centrifuge CP 80NX), followed by a separate collection of fractions from the 70% layer and the supernatant.

### 2.6. Haemagglutination Test

A haemagglutination (HA) test was performed for the H5 and H9 VLPs to determine the haemagglutinating unit (HAU) of the VLPs to formulate the dose required for the animal trials. Two-fold serial dilutions of the VLPs (50 μL) were prepared in 50 μL phosphate-buffered saline (PBS) in 96-well V-bottom microtitre plates [36]. Washed 1% chicken red blood cell suspension (50 μL) (TCS Biosciences Ltd., Buckingham, UK) was added to all wells, and the plates were incubated for 2 h at room temperature. The HAU was recorded, which is the reciprocal of the highest dilution of the VLPs showing complete haemagglutination. Plant lysate infiltrated with an empty pEAQ-HT vector was used as a negative control.

### 2.7. Transmission Electron Microscopy

Transmission electron microscopy (TEM) was used to confirm the expression of the H5 and H9 VLPs. The partially purified H5 and H9 VLPs were prepared for examination by negative-staining TEM using a JEOL 2100+TEM (Electron Microscopy Unit, nmRC, University of Nottingham). The VLPs (20 μL) were incubated for 10 min on carbon-coated copper grids (mesh size 200) and washed three times with 5 μL sterile water. Particles were negatively stained for 60 s with 2% uranyl acetate before imaging.

### 2.8. Animal Trials 

Four BALB/c mice (5 weeks old) were used (two mice each for the H5 and H9 VLPs). Each dose contained 480 HAU of VLPs and was formulated with equal amounts of TiterMax Gold (180 μL). Mice were immunised using the subcutaneous route with four doses (days 1, 15, 29 and 58). Blood samples were taken on day 0 (pre-immunisation), between the third and fourth immunisations (day 36) and at day 63 when the mice were also killed by a schedule 1 method and the spleens harvested.

As part of a larger study, six specific pathogen-free (SPF) 7-day-old chicks (eggs from Valo Biomedia) were immunised with 1024 HAU of H5 VLPs mixed with equal amounts of Montanide ISA 71 VG adjuvant (SEPPIC) (50 μL). The chicks were bled from the wing vein every week up to 6 weeks post-immunisation.

### 2.9. ELISA to Measure Antibodies

Enzyme-linked immunosorbent assay (ELISA) was performed to detect specific antibodies in mice and chicken sera. The H5 and H9 VLPs were added to each well at a concentration of 2 μg/well in 100 μL of carbonate–bicarbonate buffer (Sigma, Welwyn Garden City, UK) in a Nunc™ 96-well flat-bottom plate (ThermoFisher Scientific, Loughborough, UK). The plates were incubated at 4 °C overnight. The plates were then washed three times with 200 μL of wash buffers (PBS supplemented with 20% tween, 0.1% (*v*/*v*) and then PBS only). The blocking buffer (3% non-fat dry milk in wash buffer) was added to the wells at 200 μL/well and left at room temperature for one hour before washing. The mouse or chicken sera were added (diluted 1:1000 or 1:2000, respectively, in blocking buffer) at 100 μL/well and the plates were left at room temperature for two hours before washing, the same as before. The anti-mouse HRP-linked IgG Antibody (7076S, Cell Signaling Technology, Leiden, The Netherlands) or secondary HRP-linked goat anti-chicken IgY(H + L) antibody (6100-05, SouthernBiotech, Birmingham, AL, USA) was diluted 1:2000 or 1:3000, respectively, in blocking buffer and 100 μL/well was added. The primary and secondary antibody concentrations were optimised by titration. The plates were left for one hour at room temperature. The plates were then washed three times with wash buffers. The TMB substrate (Thermo Scientific, UK) was added at 100 μL/well and incubated for 10 min; then, 100 μL/well 2M sulphuric acid was added. A Varioskan microplate reader was used to measure the absorbance at 450 nm.

### 2.10. Pseudotyped Virus Neutralisation Test

HIV-1 lentivirus packaging construct psPAX2 encoding the gag–pol genes, and the pCSFLW lentiviral vector expressing firefly luciferase as a reporter protein (both available from Addgene) were used to generate pseudotyped viruses (PVs) expressing H5 HA. In addition, pCAGGS-HAT plasmid kindly provided by Prof Eva Böttcher-Friebertshäuser (Institute of Virology, Philipps University Marburg, Germany) was used to express the HAT protease for cleavage of the HA (Böttcher-Friebertshäuser et al., 2010). The H5 HA (clade 1) was PCR-amplified from A/Vietnam/1194/2004 (H5N1) [37], while the N8 NA was amplified from A/equine/Newmarket/5/2003 (H3N8) and was kindly provided by Prof. Nigel Temperton and Dr Simon Scott, Viral Pseudotype Unit, Medway School of Pharmacy, Kent. 

Pseudotyped viruses (PVs) were produced in human embryonic kidney (HEK) 293T cells at a density of 3 × 10^5^/well. Plasmid concentrations were measured using a Nanodrop 8000 spectrophotometer (ThermoFisher Scientific, UK). DNA mixtures were prepared containing 500 ng per well of the psPAX2 and H5 HA plasmids, 125 ng per well of N8 NA and pCAGGS-HAT plasmids and 1000 ng of pCSFLW per well. A positive control contained the psPAX2 core, pCSFLW reporter and vesicular stomatitis virus glycoprotein G (VSV-G) plasmids. A delta envelope (ΔEnv) negative control contained only the psPAX2 core and pCSFLW reporter plasmids. The DNA mixtures were made up of 100 µL of OptiMEM™ (Thermo Fisher Scientific, UK), 7 µL of lipofectamine™ 2000 (Thermo Fisher Scientific, UK) was premixed with 100 µL of OptiMEM™ and left for 5 min. The DNA mixture and the lipofectamine were combined and incubated for 15 min at room temperature before being added to the cells. The supernatant containing the PVs was harvested after 48 h of incubation at 37 °C in a 5% CO_2_ incubator, filtered using a 0.45 µm syringe filter and stored at −80 °C. The PVs were titrated and the PVNT performed following the method described by Nie et al. [38] and the Microsoft Excel file provided by the authors was used to obtain the percentage neutralisation.

### 2.11. ELISpot

Enzyme-linked immunosorbent spot (ELISpot) was used to detect interferon-γ-producing cells derived from the spleens of the immunised mice. First, 10% RPMI was prepared by adding 1% L-glutamine (Gibco^®^ Thermo Scientific™, Altrincham, UK), 100 U penicillin and 1 μg/mL streptomycin (Gibco^®^ Thermo Scientific™, Altrincham, UK), 10% foetal calf serum (Sigma, UK) and 1% HEPES solution (Sigma, UK) to Roswell Park Memorial Institute media (Gibco^®^ Thermo Scientific™, Altrincham, UK). The spleens were squeezed using a syringe containing 20 mL of 10% RPMI to harvest the splenocytes and the cell suspension was centrifuged at 300× *g* for 10 min. The cell pellet was resuspended in 10 mL of 10% RPMI medium, which also contained 50 μM of 2-mercaptoethanol, and the cells were counted in a haemocytometer (typically around 5 × 10^6^ cells/mL). The precoated ELISpot Plus: Mouse IFN-γ (ALP) 96-well plates (Mabtech, Stockholm, Sweden) were incubated at room temperature for 30 min with 10% RPMI media without 2-mercaptoethanol before the media were discarded. Then, 100 μL per well of the cells was added. Concentrations of the H5 or H9 VLPs ranging from 0.1 to 2.3 μg/mL) were prepared in 10% RPMI containing 50 μM of 2-mercaptoethanol, and 100 μL of these VLPs were added to duplicate wells containing splenocytes. Concanavalin A (con A) (Invitrogen, Thermo Fisher Scientific, UK) at 0.5 μL per ml of RPMI media containing 50 μM of 2-mercaptoethanol (final concentration 0.625 ng/mL) was added to duplicate wells as a positive control. Two negative controls were used: the cell-only control and the negative control lysate (plants infiltrated with empty pEAQ-HT vector). The plates were incubated at 37 °C for 48 h in a 5% CO_2_ incubator. Then, the cells were flicked off the plates, and the experiment was developed according to the manufacturer’s protocol. The spots in each well were detected and counted using an automated plate reader (Cellular Technology Ltd., Cleveland, OH, USA).

## 3. Results

### 3.1. Expression of H5 and H9 VLPs in Plants

**Figure 1:**
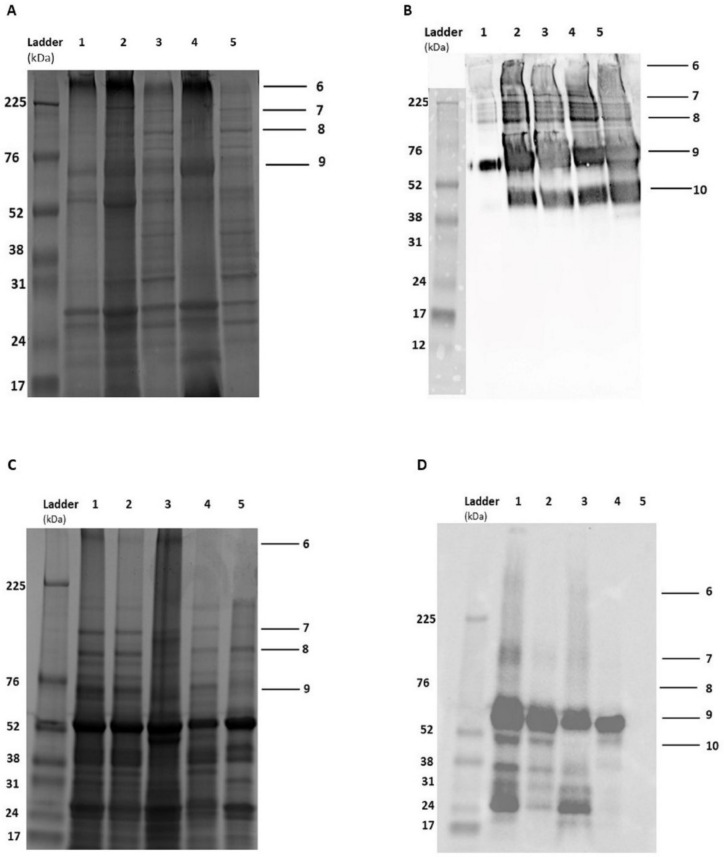
Purification of H5 and H9 VLPs using sucrose cushion ultracentrifugation. Panels (**A**) and (**B**) illustrate the stained SDS-PAGE gel and western blot of the H9 VLP purification, respectively, while panels (**C**,**D**) illustrate the stained SDS-PAGE gel and western blot of the H5 VLP purification, respectively. Panels (**A**,**B**), lane 1: H9 pellet, lane 2: H9 plant lysate, lane 3: syringe-filtered H9 plant lysate, lane 4: concentrated H9 VLPs and lane 5: collected supernatant after ultracentrifugation. Panels (**C**,**D**), lane 1: H5 plant lysate, lane 2: syringe-filtered H5 plant lysate, lane 3: concentrated H5 VLPs, lane 4: collected supernatant after ultracentrifugation and lane 5: negative control plant lysate infiltrated with an empty pEAQ-HT vector. Bands (6–9) represent the H9 VLP, trimer, dimer and monomer patterns, respectively. A smaller band (10) is suggestive of some protein degradation.

H5 and H9 VLPs were transiently expressed in *N. benthamiana* using syringe infiltration. Expression was confirmed using western blot analysis and TEM. The H5 and H9 VLPs were partially purified and concentrated using sucrose density cushion ultracentrifugation. The purification process was assessed using stained sodium dodecyl sulphate–polyacrylamide gel electrophoresis (SDS-PAGE) gels under reducing conditions. The H9 VLPs showed bands with a molecular weight of approximately 66, 170 and 225 kDa, corresponding to a monomer, dimer and trimer of HA, respectively (Figure 1A,B). The H5 VLPs showed the same bands as the H9 VLPs, except that the monomer was around 67 kDa (Figure 1C,D). A major band was detected near the top of the gel corresponding to the VLPs. Minor bands were detected below 52 kDa by western blot, presumably due to degradation of proteins during the extraction process. Figures S1–S4 in the supplementary materials present the original images of SDS-PAGE and western blot. 

**Figure 2:**
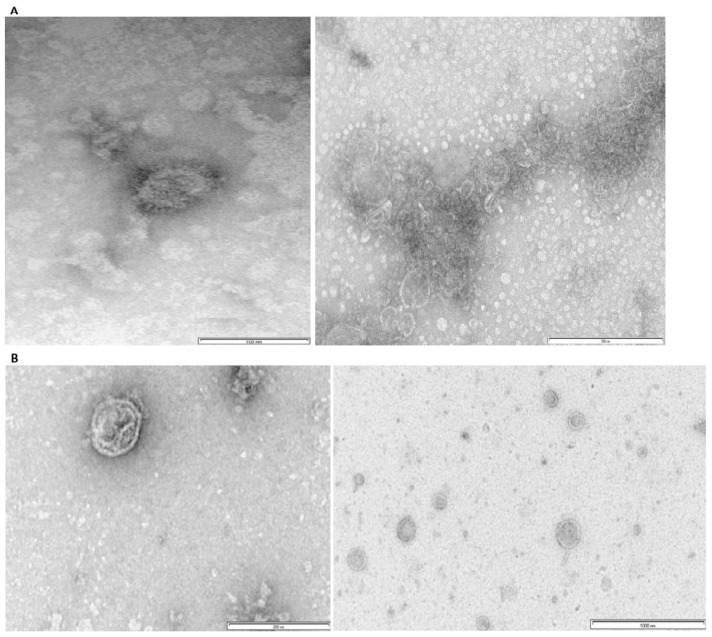
Negatively stained TEM of the H5 and H9 VLPs. Figures (**A**,**B**) represent the H9 and H5 VLP, respectively. The VLPs measure around 100 nm.

Negative-staining TEM confirmed the presence of the H5 and H9 VLPs in the partially purified plant lysate. The VLPs were spherical, and their size was around 100 nm, resembling the native influenza virus particle (Figure 2). Figures S4–S8 in the supplementary materials show the original images of TEM.

A HA test was used to confirm the integrity of HA in the VLP preparations and determine the HAU to calculate the dose required for vaccination in animal trials. The H5 and H9 VLPs showed titres of around 512 and 256 HAU per 50 μL, respectively. Negative control plant lysate infiltrated with empty pEAQ-HT showed no agglutination of the red blood cells of chickens.

### 3.2. Mouse Immunisation Study

**Figure 3:**
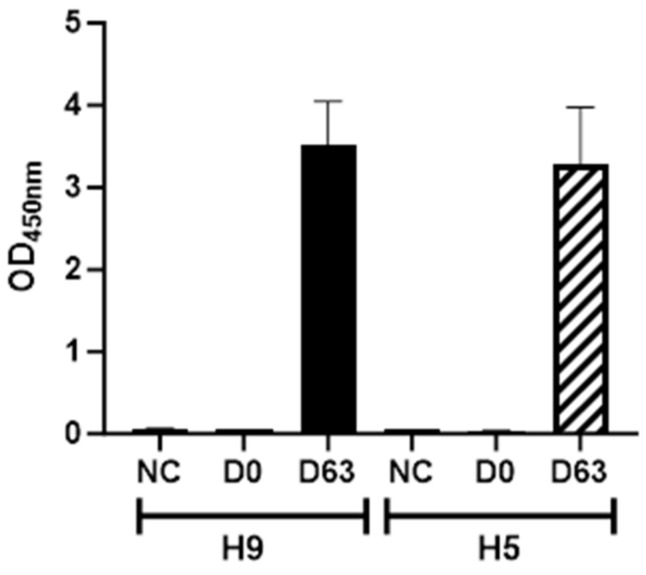
ELISA using H5 and H9 virus-like particles (VLPs) and pooled mice sera. Pre-immunisation sera sampled at day 0 and post-immunisation sera sampled at day 36 and day 63 were pooled from each pair of mice immunised with either H5 or H9 VLPs and diluted 1:1000. Pre-immunisation samples were added to wells of an ELISA plate coated with either H5 or H9 VLPs and post-immunisation sera samples were added to wells coated with either H5 or H9 VLPs or negative control plant lysate (NC). Mean OD at 450 nm for duplicate wells (±SEM) are shown.

ELISA was performed to detect HA-binding antibodies in the sera from immunised mice, which were pooled due to the small volumes obtained. High OD values were obtained with pooled post-immunisation sera from both H5 VLP and H9 VLP-immunised mice (Figure 3). The negative control plant lysate containing an empty pEAQ-HT vector showed negligible reactivity with H5 and H9 antisera.

**Figure 4:**
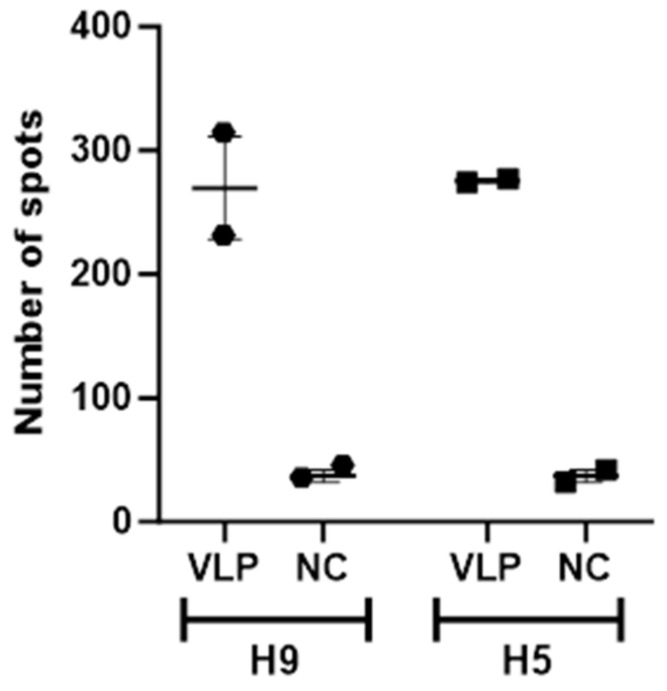
Interferon-γ ELISpot results for spleen cells restimulated with either H5 or H9 VLPs or negative control plant lysate (NC) at 0.9 µg/mL. Cell-only control wells had a maximum of 6 spots. The results were calculated as mean (±SEM) of duplicate wells.

ELISpot assays were performed to detect interferon-γ secreting splenocytes in mice immunised with H5 and H9 VLPs. The optimum concentration of VLPs for restimulation of the splenocytes was determined to be around 0.9 µg/mL (fewer spots were produced at higher concentrations). Restimulation of splenocytes with H5 and H9 VLPs showed a high number of spots with a mean ±SEM of 254.5 for H5 and 275.5 for H9 VLPs (Figure 4). Restimulation with negative control plant lysate from plants infiltrated with empty pEAQ-HT showed few spots, while the cell control wells had fewer than six spots.

### 3.3. Chicken Immunisation Study

**Figure 5:**
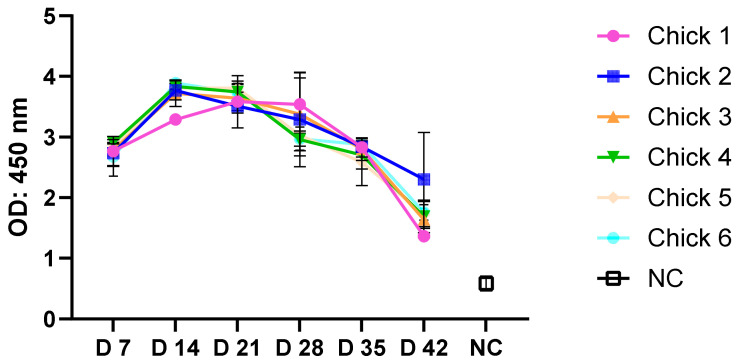
ELISA using H5 VLPs and sera from six chickens immunised with the H5 VLP vaccine. The negative control plant lysate derived from plants infiltrated with *Agrobacteria* containing an empty pEAQ-HT vector showed negligible reaction with an OD at 450nm of around 0.5. The results were calculated as mean (±SEM) of duplicate wells.

An ELISA was performed to detect binding antibodies to H5 VLP in chicken sera following immunisation. All sera showed evidence of binding antibodies at day 7 post immunisation with an OD of around 2.7. Binding antibody titres reached the maximum at day 14 and decreased gradually to a mean OD of 1.7 at day 42 (Figure 5). 

A negative control of plant lysate derived from infiltration of plants with an empty pEAQ-HT vector showed negligible reaction with immunised H5 sera, with an OD of around 0.5.

**Figure 6:**
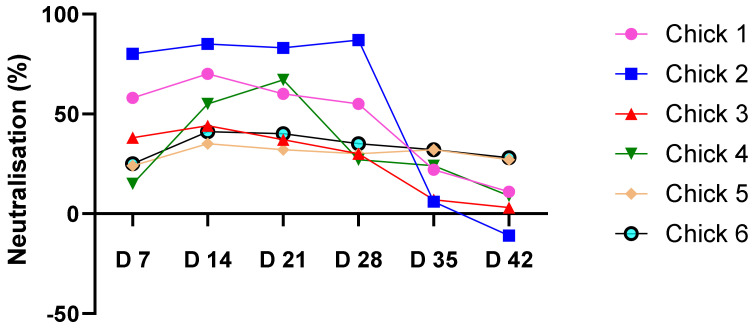
Percentage of neutralisation obtained with A/Vietnam/1194/2004 (H5N1) pseudotyped virus VNT with sera from six chickens immunised with the A/chicken/Egypt/FL6/2018 (H5N8) H5 VLP vaccine. The mean percentage neutralisation of negative chicken sera was −0.3%. The percentage of neutralisation from duplicate wells was calculated using the Excel sheet supplied by Nie et al. [35].

PVNT was used to detect HA-neutralising antibodies in sera from H5 VLP immunised chickens. HA-neutralising antibodies were detected at day 7 (mean = 40%), reaching maximum levels by day 14 (55%), and decreased gradually to a mean value of 11% at day 42 (Figure 6). The response varied between animals, with three chickens showing a titre of over 50% (1, 2 and 4) while three animals showed neutralisation of 30–40% following the single immunisation with adjuvanted H5 VLP. The highest percentage of neutralisation was observed in chicken 2 at day 28 post-immunisation (87%). The negative chicken sera did not show any neutralisation (−0.33%) with H5 PVs.

## 4. Discussion

In this project, both H9 and H5 VLPs were successfully expressed in *N. benthamiana*. Western blot confirmed the presence of H5 and H9 proteins using specific H5 and H9 antibodies, respectively. Multiple bands were evident, suggesting that HA dimers and trimers were present in addition to monomers. Larger molecular-weight bands were also present, supporting the formation of VLPs. Castells-Graells and Lomonossoff [39] suggested that plant expression using *N. benthamiana* and *Agrobacterium* can result in the cross-linking of protein forming higher molecular-weight structures resistant to denaturation. They suggested that some cross-linking of subunits could be beneficial for the stability of VLP vaccines. For detailed information, see Figure S9 in the supplementary materials. 

The VLPs were partially purified using sucrose cushion ultracentrifugation and negative staining by TEM confirmed the presence of the H5 and H9 virus-like particles that were spherical and measured around 100 nm. The TEM results for H9 and H5 VLPs agree with the previous reports of producing H5 VLPs [40,41], H7 VLPs [42] and H6 VLPs [43]. The measurement of HA titre was used to quantify the VLPs for animal studies. The H5 and H9 VLPs had similar titres of 1:512 and 1:256, respectively.

In a previous study, it was suggested that co-expression of matrix protein (M1) may be essential for the formation of the VLPs [44] and the stability of the influenza VLPs [43]. However, the HA alone was expressed in this study, which agrees with the results of [40] supporting the ability of HA alone to generate influenza VLPs. Nonetheless, there was evidence of some degradation of VLPs in the Western blots, and the inclusion of M1 might better stabilise the incorporation of HA into the VLPs. Codon optimisation of the desired gene is usually performed for higher expression levels. H5 and H9 VLPs were codon-optimised to reflect the codon usage of the *N. benthamiana* and a high VLP yield was detected. While codon optimisation is often performed to enhance heterologous protein expression, the effect varies according to the gene and the expression system used [45,46]. A Kozak sequence was added upstream of the start codon in the VLPs and was not expected to negatively affect the VLP expression. However, more studies are needed to compare the effect of the Kozak sequence on the VLP expression. Previous studies have reported that the Kozak sequence enhanced translation in eukaryotic cells and plants [47,48,49]. The HA native signal peptide was replaced by PR1a tobacco signal peptide to ensure high expression levels in the plant cells in accordance with previous studies [49,50,51] that replaced the native signal peptide with PR1a signal peptide. The VLPs were only partially purified but did not elicit any adverse effects in the immunised mice and chickens in our study, which agrees with a previous study in which mice were immunised with recombinant HA crude plant extract without side effects [23]. The ability to use partially purified proteins without adverse effects may offer an advantage for the use of plant-expressed VLP vaccine in resource-poor countries.

Animal models such as mice, guinea pigs and ferrets are frequently used to assess the immunogenicity of vaccines [52,53,54]. Indirect ELISA using pooled immunised H5 and H9 mice sera showed high HA binding antibodies against H5 and H9 VLPs, respectively. We are not aware of plant-expressed VLPs being used previously to restimulate spleen cells for ELISpot. The amount of plant-expressed VLP required to restimulate spleen cells from mice was optimised and the background obtained with the negative control plant lysate was low. Typically, restimulation of cells using a pool of antigen-derived peptides gives a better response than using recombinant protein. However, a high number of interferon-γ secreting splenocytes, isolated from mice immunised with H9 and H5 VLPs, were detected after restimulation with H9 and H5 VLPs using the ELISpot technique. In future studies, it would be interesting to restimulate splenocytes from H5 VLP-immunised mice with a range of antigenically diverse stimuli to characterise the breadth of the cellular immune response. In one study, mice received three doses of plant-based H5 recombinant proteins that elicited reasonable interferon-γ secreting splenocytes but poor HA-specific antibodies [55]. Recombinant HA proteins expressed using a baculovirus vector in insect cells showed a broad protective immune response in mice. The limitation of this system is that although VLPs are more immunogenic, purification of the VLPs from the unwanted vector proteins is expensive and time-consuming [56]. In other studies, AIV VLP elicited cellular and humoral immune responses using low doses in humans [30] or mice and ferrets [57].

Chickens are the ideal model for testing the immunogenicity of AIV vaccines because they are the target host [58]. However, more commercial reagents are available for measuring murine immune responses, including ELISpot kits, which can enumerate IFN-γ secreting cells as an indicator of an antigen-specific T-cell response. Therefore, the VLPs were initially screened for immunogenicity in mice. A hyper-immunisation schedule was used to enable subsequent production of monoclonal antibody-producing hybridoma cells. It would be desirable to determine cellular immune response in chickens, and assays optimised for this purpose are still being developed [59].

Chicken immunisation with a single dose of H5 VLPs elicited high levels of anti-HA antibodies detected by ELISA. The immune response began to be detected from day 7, then increased until the maximum was reached on day 14, and started to decrease gradually until the lowest titre was reached by day 42. Chickens immunised with plant-based H5 recombinant proteins showed fewer specific anti-HA antibodies compared to our results by ELISA [60,61]. Plant-based H5 oligomer proteins elicited slightly lower HA-specific antibodies in chickens than our VLPs [23]. Generally, the literature shows that VLP vaccines generate an excellent immune response compared to recombinant proteins due to proper antigen presentation to the relevant immune cells. Therefore, unlike recombinant proteins, VLPs may not need an adjuvant to improve the immune response [27,28,29]. In future studies, it would be interesting to compare immunisation with VLPs with and without the adjuvant used in this study and compare the responses to recombinant (subunit) HA vaccines.

As far as we are aware, a PVNT was used here for the first time to assess the neutralising antibody response of chickens to a plant-based vaccine. The PVNT offers a safe platform for measuring the neutralising immune response generated by vaccination [38,62,63]. PVNT, therefore, offers an alternative to animal challenge studies where the antibody titre required for protection is known. Chickens receiving a single dose of H5 VLPs showed a mean peak neutralising antibody response of 55% on day 14 post-immunisation. However, it should be noted that the HA sequence used in the pseudotyped virus was heterologous (clade 1) to the sequence used in the VLPs (clade 2.3.4.4b).

A booster dose is recommended for immunised chickens with H5 VLPs for higher binding and neutralising antibody responses. In the future, the minimal protective dose of the VLP vaccines should be evaluated in chickens, and protection against homologous and heterologous virus strains should be evaluated by challenging the immunised chickens with the relevant virus strains. Further studies may also allow protective antibody levels measured by PVNT to be determined, and this method of measurement could be used as an alternative to animal challenge studies in the future.

## 5. Conclusions

Plant-based H9 and H5 VLPs expressed in *Nicotiana benthamiana* demonstrated potential for use as effective and cheap vaccines for avian influenza, which may be especially valuable for countries with fewer resources.

## Data Availability

Data are contained within the article and Supplementary Materials.

## Supplementary Materials

https://www.mdpi.com/article/10.3390/vetsci11020093/s1, Figure S1: Stained SDS-PAGE gel of the H9 VLPs purification using sucrose cushion ultracentrifugation. Figure S2: Western blot of the H9 VLPs purification using sucrose cushion ultracentrifugation. Figure S3: Stained SDS-PAGE gel of the H5 VLPs purification using sucrose cushion ultracentrifugation. Figure S4: Western blot of the H5 VLPs purification using sucrose cushion ultracentrifugation. Figures S5 and S6: Negatively stained TEM of H9 VLPs. Figures S7 and S8: Negatively stained TEM of H9 VLPs. Figure S9: Western blot of the purification of pEAQ-HT-H9-SUMO-tag and pEAQ-HT-H5-SUMO-tag protein using tetra His-tag antibody.
