# Supplementary material for: Development of Virus-like Particle Plant-Based Vaccines against Avian H5 and H9 Influenza A Viruses
Source: Vet Sci. 2024 Feb 18;11(2):93. doi: 10.3390/vetsci11020093 (PMC10891754; doi:10.3390/vetsci11020093)
Supplement: Supplementary file 1 [file vetsci-11-00093-s001.zip › vetsci-2837101-supplementary.pdf]

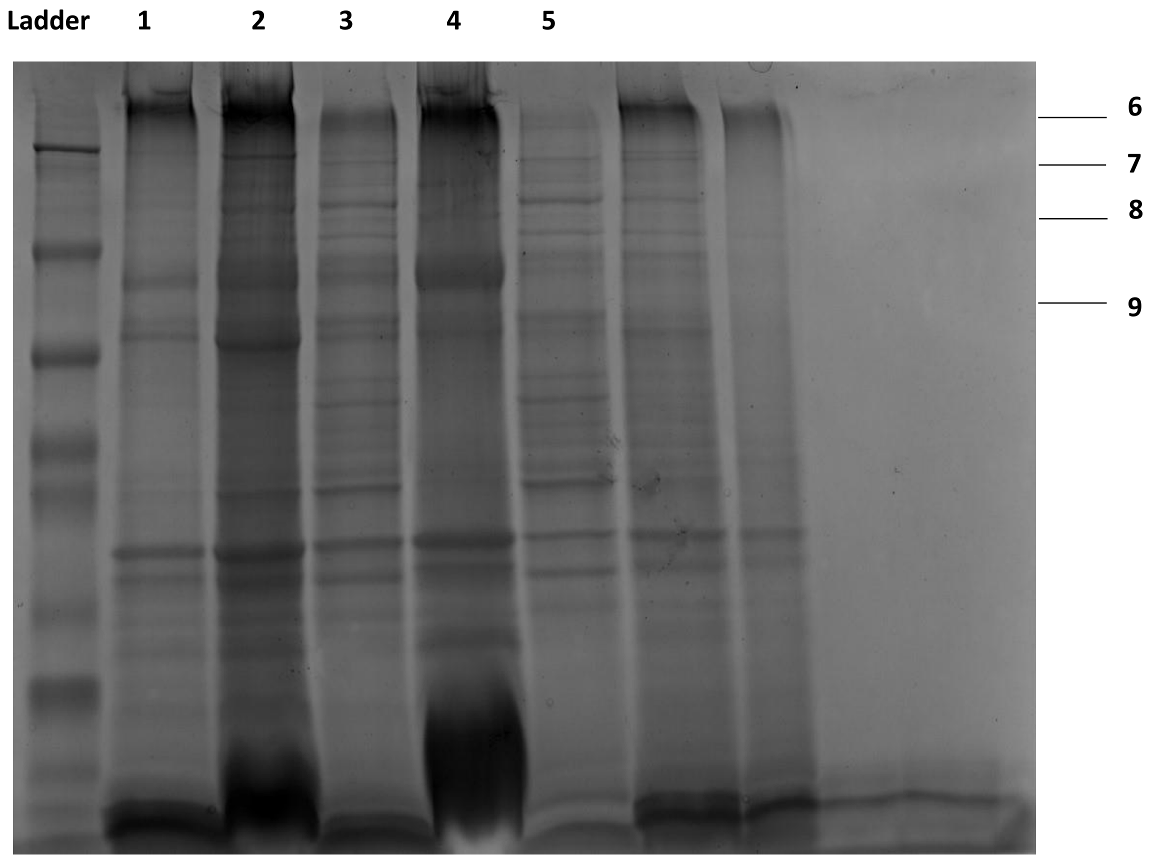

Figure S1. Stained SDS-PAGE gel of the H9 VLPs purification using sucrose cushion ultracentrifugation. Lane 1: H9 pellet, lane 2: H9 plant lysate, lane 3: syringe-filtered H9 plant lysate, lane 4: concentrated H9 VLPs, and lane 5: collected supernatant after ultracentrifugation. Bands (6–9) represent the H9 VLP, trimer, dimer, and monomer patterns, respectively.

Note: It was cropped after lane 5 because the remaining samples belonged to another experiment that was not discussed in the manuscript.

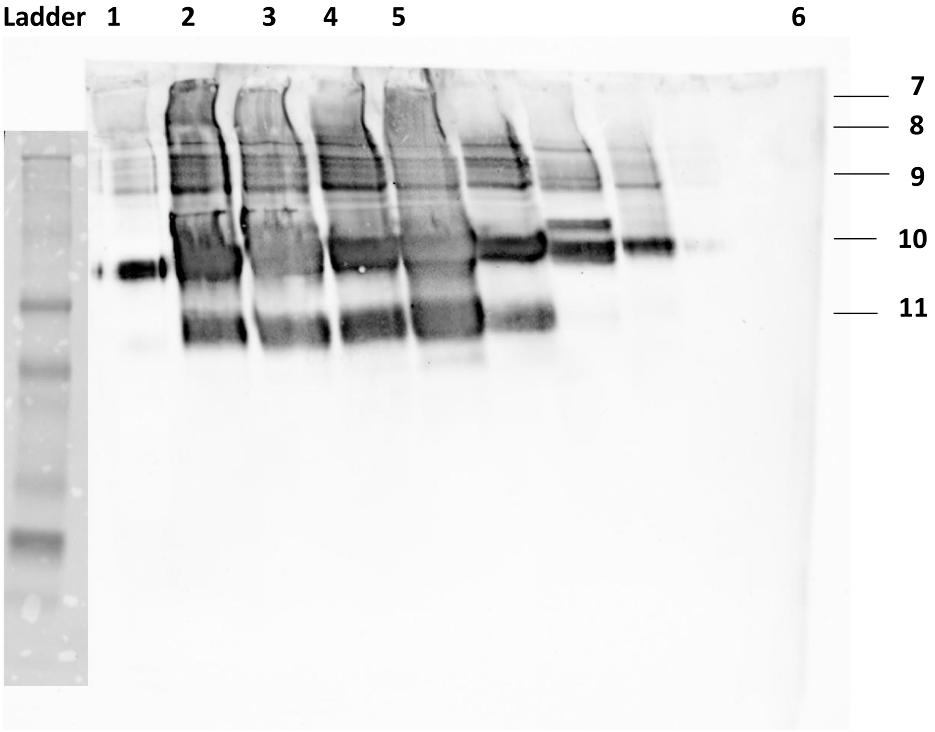

Figure S2. Western blot of the H9 VLPs purification using sucrose cushion ultracentrifugation. Lane 1: H9 pellet, lane 2: H9 plant lysate, lane 3: syringe-filtered H9 plant lysate, lane 4: concentrated H9 VLPs, and lane 5: collected supernatant after ultracentrifugation, and lane 6: negative control plant lysate infiltrated with empty pEAQ-HT vector. Bands (7-10) represent the H9 VLP, trimer, dimer, and monomer patterns, respectively. A smaller band (11) is suggestive of some protein degradation.

Note: It was cropped after lane 5 because the remaining samples belonged to another experiment that was not discussed in the manuscript. Lane 6 is a negative control plant lysate infiltrated with an empty pEAQ-HT vector.

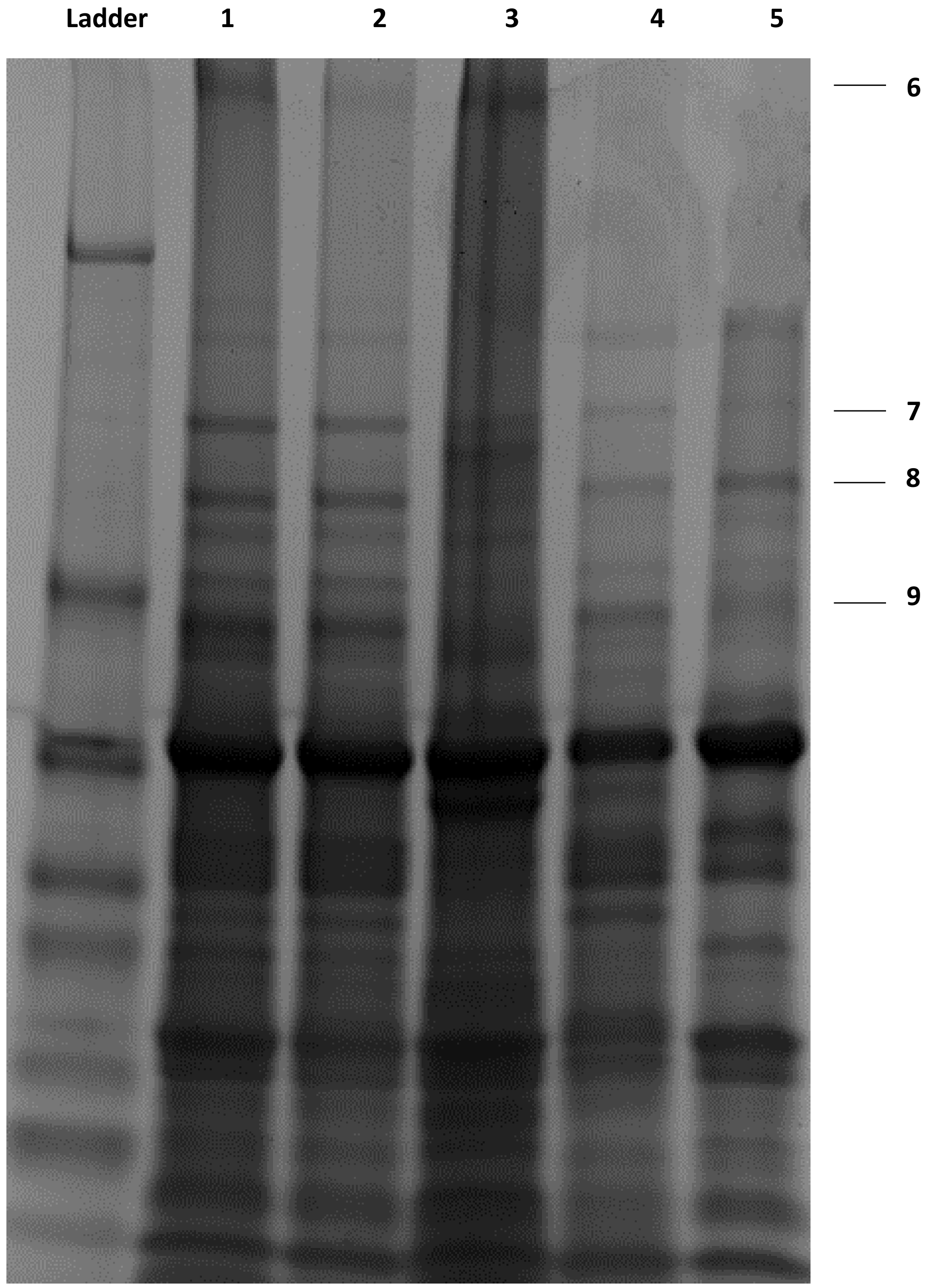

Figure S3. Stained SDS-PAGE gel of the H5 VLPs purification using sucrose cushion ultracentrifugation. Lane 1 H5 plant lysate, lane 2: syringe-filtered H5 5 plant lysate, lane 3: concentrated H5 5 VLPs, and lane 4: collected supernatant after ultracentrifugation, and lane 5: negative control plant lysate infiltrated with empty pEAQ-HT vector. Bands (6–9) represent the H9 VLP, trimer, dimer, and monomer patterns, respectively.

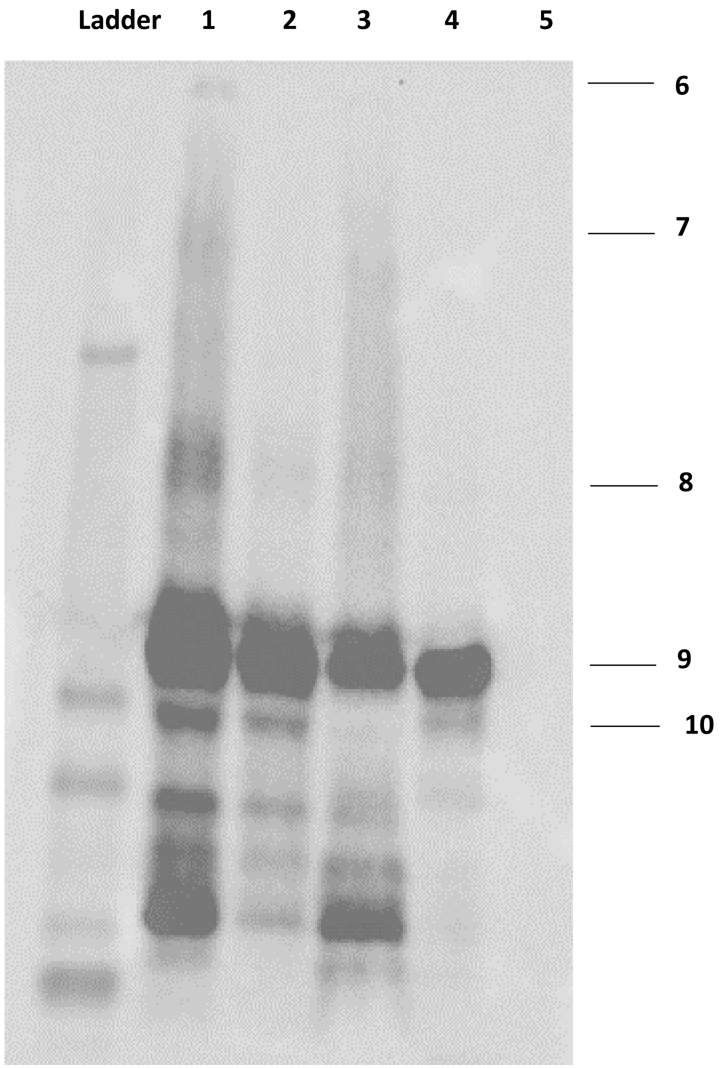

Figure S4. Western blot of the H5 VLPs purification using sucrose cushion ultracentrifugation. Lane 1 H5 plant lysate, lane 2: syringe-filtered H5 5 plant lysate, lane 3: concentrated H5 5 VLPs, and lane 4: collected supernatant after ultracentrifugation, lane 5: negative control plant lysate infiltrated with empty pEAQ-HT vector. Bands (6–9) represent the H9 VLP, trimer, dimer, and monomer patterns, respectively. A smaller band (10) is suggestive of some protein degradation.

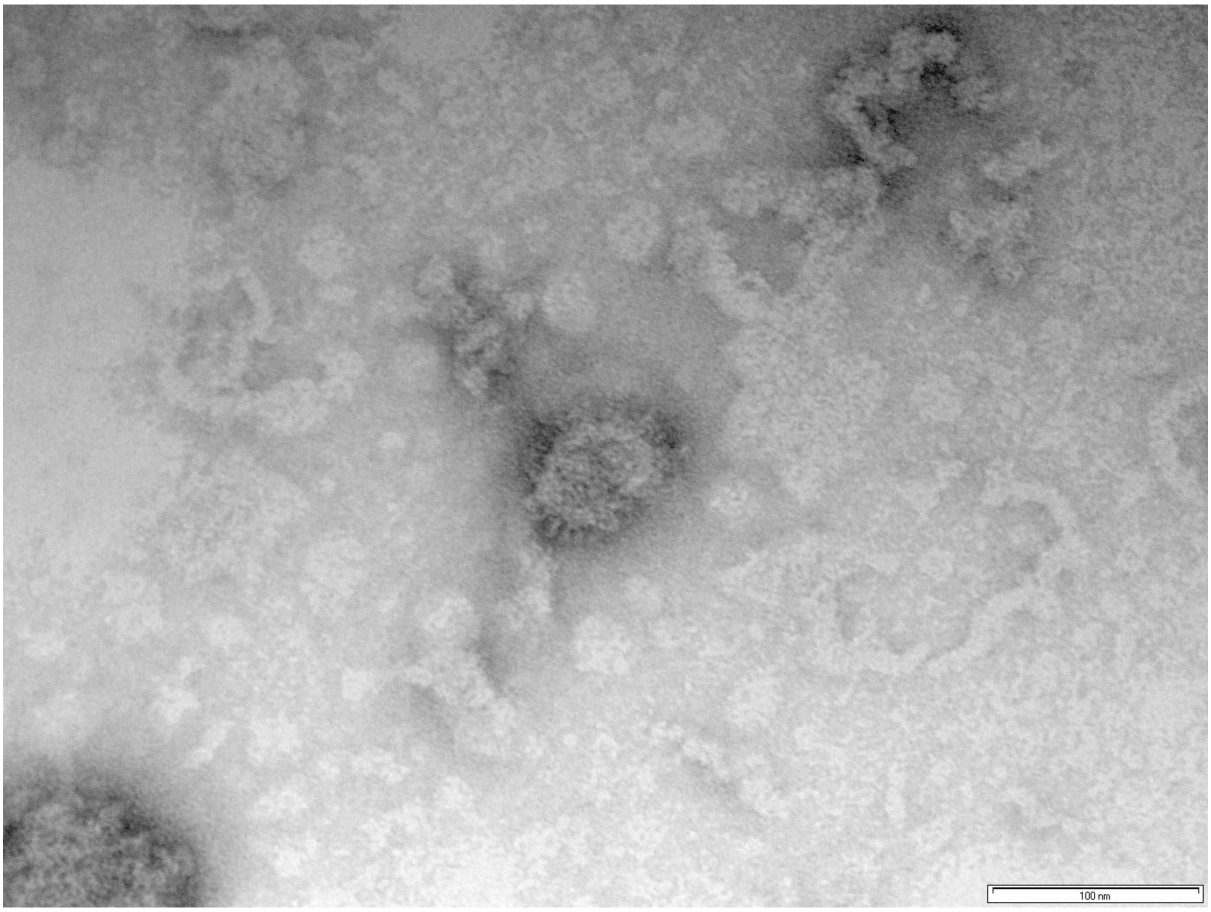

Figure S5. Negatively stained TEM of H9 VLPs. Figure A represents the H9 VLP. The VLPs measure around 100 nm.

Note: it was cropped to show the VLP structure.

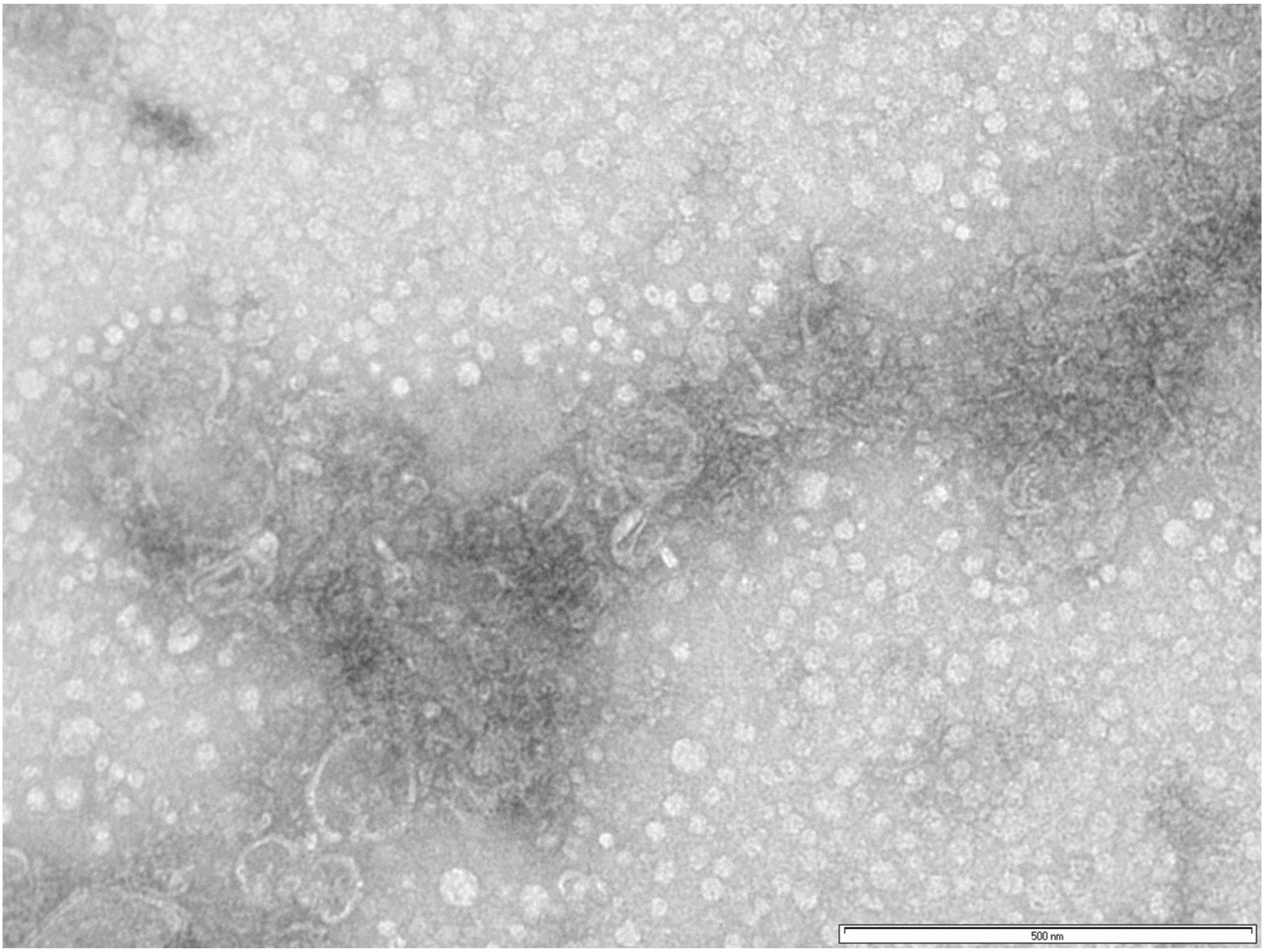

Figure S6. Negatively stained TEM of H9 VLPs. Figure A represents the H9 VLP. The VLPs measure around 100 nm.

Note: it was cropped to show the VLP structure.

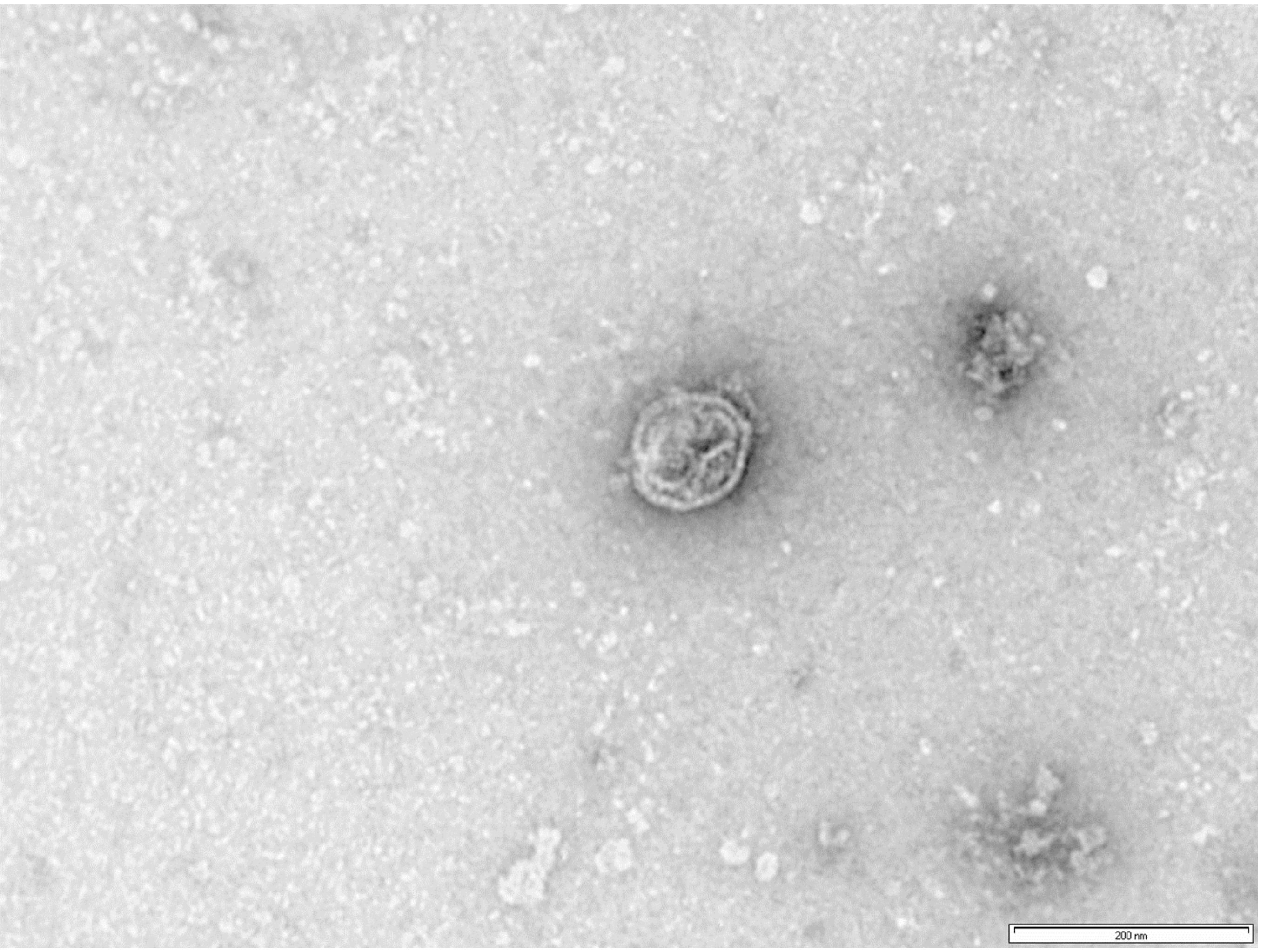

Figure S7. Negatively stained TEM of H5 VLPs. Figure B represents the H5 VLP. The VLPs measure around 100 nm.

Note

Note: it was cropped to show the VLP structure.

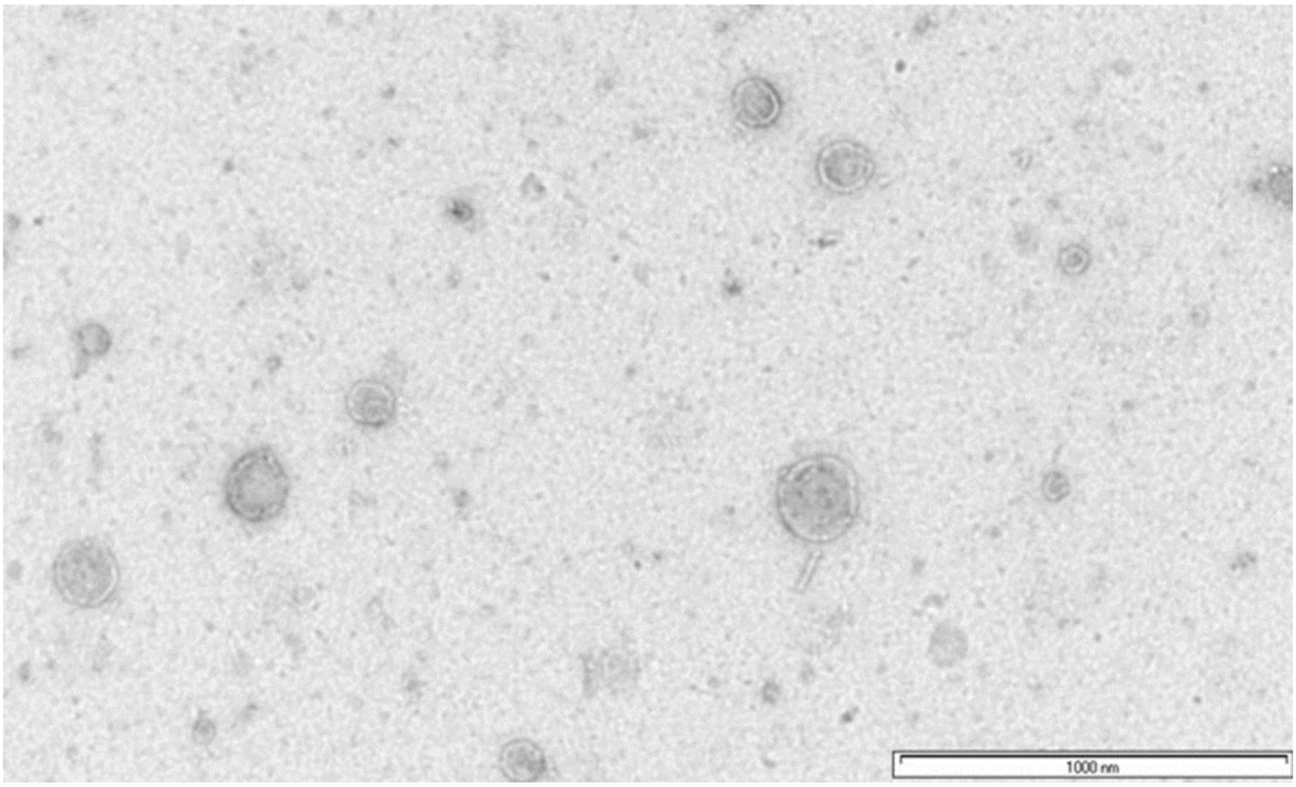

Figure S8. Negatively stained TEM of H5 VLPs. Figure B represents the H5 VLP. The VLPs measure around 100 nm.

Note: it was cropped to show the VLP structure.

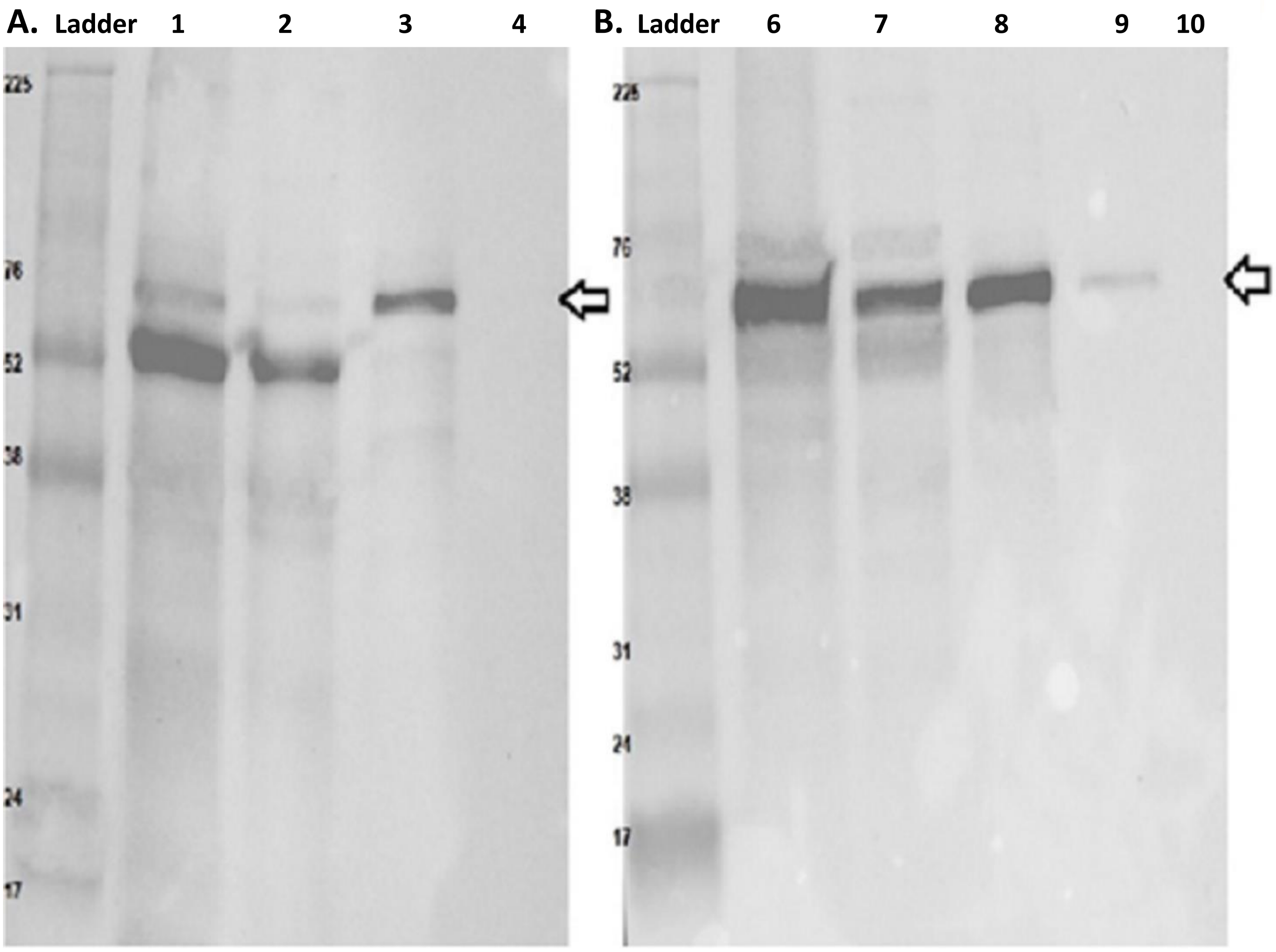

Figure S9. Western blot of the purification of pEAQ-HT-H9-SUMO-tag and pEAQ-HT-H5-SUMO-tag protein using tetra His-tag antibody. Figure A. Lane 1 represents the H9 plant lysate, Lane 2 is the H9 flow-through, Lane 3 is the first elution, and Lane 4 is the negative control lysate. The H9-SUMO-tagged proteins showed the band at the expected size of around 72.6 kDa, as represented by the arrow. Figure B. Lane 5 is the H5 plant lysate, lane 6 is the H5 flow-through, lane 7 is the first wash, lane 8 is the first elution, lane 9 is the second elution, and lane 10 is the negative control lysate. The H5-SUMO-tagged proteins showed the band at the expected size of around 73.7 kDa, as represented by the arrow.

Note. pEAQ-HT-H5-SUMO-tag and pEAQ-HT-H9-SUMO-tag proteins are the H5 and H9 recombinant proteins after removing the TMD. Western blot was performed using the same reducing SDS-PAGE gel, which was used for detecting the VLPs. Dimer and trimer patterns could not be detected.
